# OMS-led Education Improves Adolescent Marijuana and Vaping Health Literacy Across Multiple Cohorts

**DOI:** 10.51894/001c.164400

**Published:** 2026-08-01

**Authors:** Andrew Saad, Anushree Ravi, Carrie Nazaroff, Carolina Restini

**Affiliations:** 1 College of Osteopathic Medicine, Michigan State University

## 47

### Introduction

Adolescent substance use has intensified following marijuana legalization and the emergence of electronic nicotine devices. Marijuana use during adolescence is associated with impaired neurodevelopment and increased academic risk. In parallel, high-nicotine vaping devices have contributed to youth nicotine dependence and increased risk of future substance use disorders. In response, the Substance Use Prevention (SUP) program was established in 2019 at MSUCOM, using a near-age peer model led by osteopathic medical students (OMS) to deliver interactive, evidence-based prevention education.

### Objectives

The primary objective was to evaluate the cumulative impact of SUP across cohorts. We assessed changes in marijuana knowledge and vaping confidence across implementation years. We hypothesized that OMS-led education would produce significant improvements with stable effect sizes across cohorts despite evolving secular trends.

### Methods

We conducted a retrospective analysis of SUP interventions delivered by OMS across Michigan schools from 2024-2026 (IRB approval:8018). Pre-/post-surveys assessed marijuana knowledge and vaping confidence. Knowledge was based on construct aggregation, while confidence was measured with a standardized five-point Likert scale. Paired analyses evaluated pre-post change and cohort differences.

### Results

Across cohorts, students demonstrated significant improvements in their confidence about information regarding vaping. In 2024 (n=50), scores increased from 2.64 to 3.70 (+1.06) (*p* < 0.001). In 2025 (n= 100), scores increased from 2.64 to 3.73 (+1.09) (*p* < 0.001). In 2026 (n=158), scores increased from 2.81 to 3.91 (+1.10) (*p* < 0.001). Similarly, students demonstrated significant improvements in marijuana knowledge. In 2025 (n=50), knowledge increased 21%, and in 2026 (n=158) increased 29.5% (both *p* < 0.05). No between-year difference (*p* = 0.24). ANOVA of change scores showed no difference across cohorts (*p* = 0.96), indicating stable effect sizes across successive SUP leadership cohorts.

### Conclusions

Longitudinally, the OMS-led near-age education model consistently produced significant improvements in marijuana knowledge and vaping confidence. Gains remained large and comparable across cohorts. Stable effect sizes support scalability and sustainability across diverse school settings. Consistent gains across years suggest a consistent educational impact despite evolving secular trends. SUP serves as a bridge between healthcare trainees and at-risk youth through community engagement. Embedded within a CBPR framework, SUP advances competency-based medical education by developing OMS skills in research and public health advocacy.

